# Acute kidney injury due to glomerular haematuria and obstructive erythrocyte casts associated with thrombocytopaenia and thin basement membrane disease: a case report

**DOI:** 10.1186/s12882-015-0176-4

**Published:** 2015-10-30

**Authors:** Andy K. H. Lim, Susan Brown, Ian Simpson, John P. Dowling

**Affiliations:** Department of Nephrology, Monash Medical Centre, 246 Clayton Road, Clayton, VIC 3168 Australia; Department of Haematology, Monash Medical Centre, 246 Clayton Road, Clayton, VIC 3168 Australia; Department of General Medicine, Monash Medical Centre, 246 Clayton Road, Clayton, VIC 3168 Australia; Department of Anatomical Pathology, Monash Medical Centre, 246 Clayton Road, Clayton, VIC 3168 Australia; Department of Medicine, Monash University, Monash Health, 246 Clayton Road, Clayton, VIC 3168 Australia

**Keywords:** Acute kidney injury, Thrombocytopaenia, Haematuria, Erythrocyte casts, Thin basement membrane disease, Chronic kidney disease

## Abstract

**Background:**

Acute kidney injury due to glomerular bleeding has been described with IgA nephropathy and supratherapeutic warfarin anticoagulation. There is usually demonstrable tubular obstruction by erythrocyte casts associated with acute tubular injury. Although severe thrombocytopaenia increases the risk of bleeding, most cases of haematuria have been ascribed to non-glomerular or urological bleeding without a direct link to acute kidney injury. We describe a patient with acute kidney injury due to glomerular bleeding and tubular injury related to severe thrombocytopaenia, who was subsequently found to have thin basement membrane disease.

**Case presentation:**

A 56 year old man presented with macroscopic haematuria, acute kidney injury and a platelet count of 35 × 10^9^/L, in the absence of anticoagulation. Urinalysis demonstrated an active urinary sediment. His kidney biopsy demonstrated extensive intraluminal erythrocyte casts associated with acute tubular injury, along with haemosiderin deposition suggestive of recurrent glomerular bleeding. There was no histological evidence of glomerular pathology but electron microscopy analysis demonstrated thin basement membrane disease and effacement of podocyte foot processes. During long term follow-up, thrombocytopaenia and intermittent haematuria persisted. At 9 months, the patient progressed to Stage 5 chronic kidney disease with the development of gross renal atrophy.

**Conclusion:**

Recurrent macroscopic haematuria may be a risk factor for progressive renal injury in patients with thin basement membrane. The mechanism may be due to recurrent acute kidney injury from glomerular bleeding leading to repeated tubular damage. In the absence of anticoagulation, severe thrombocytopaenia may be a risk factor for heavy glomerular bleeding and acute kidney injury in these patients.

## Background

The development of acute kidney injury (AKI) due to glomerular haemorrhage has been described in patients with IgA nephropathy and warfarin over-anticoagulation [1, 2]. The latter has been termed warfarin-related nephropathy. Patients often report macroscopic haematuria and the mechanism of injury is thought to be intraluminal tubular obstruction by erythrocyte casts and toxic injury from haemoglobin breakdown products [1]. Severe thrombocytopenia has also been associated with haematuria but the majority of reports indicate a non-glomerular (urological) origin which does not directly cause AKI. We report a patient who developed AKI in the setting of glomerular haematuria precipitated by thrombocytopenia, who was subsequently found to have thin basement membrane disease.

## Case presentation

A 56-year old man presented to a regional hospital with acute gout of the right knee, on a background of 6 months of lethargy, easy bruising, epistaxis and recent episodes of macroscopic haematuria. On presentation, he was noted to have AKI, anaemia, thrombocytopaenia and abnormal liver function tests. His past medical history included gout, alcoholism (10 standard drinks daily), gastro-oesophageal reflux and previous gastric fundoplication. He was not on any regular medications. He lived alone and was an active smoker of 20 pack-years but denied intravenous or recreational drugs. He reported no fever, sweats or weight loss (body mass index, 22.9 kg/m^2^). He had no haemodynamic compromise and was transferred to our centre for further management.

On arrival, his blood pressure was 140/80 mmHg. He had a normal mental status and no focal neurological signs. Physical examination was notable for mild hepatomegaly, subcutaneous bruising and spider naevi. Macroscopic haematuria was evident when the patient voided. His vital signs were normal and the laboratory results on arrival are shown in Table 1. The blood film showed target cells, anisocytosis, stomatocytes, occasional myelocytes and confirmed thrombocytopaenia. In retrospect, thrombocytopaenia was first noted eight months prior to presentation (platelet count, 60 × 10^6^/L; reference range [RR], 150–400 × 10^6^/L). At this time, the patient’s serum creatinine was 100 μmol/L (RR, 60–110 μmol/L), with an estimated glomerular filtration rate (eGFR) of 73 ml/min. Kidney function was noted to be slightly impaired (serum creatinine, 113 umol/L (eGFR, 63 ml/min) four months ago, after the onset of haematuria was noted.

**Table 1:** Laboratory parameters

Parameter	Patient value	Reference
Haemoglobin (g/L)	73	130–180
White cells ( × 10^9^/L)	5.8	4.0–11.0
Plateletes ( × 10^9^/L)	35	150–450
Neutrophils ( × 10^9^/L)	2.93	2.00–8.00
Lymphocytes ( × 10^9^/L)	1.83	1.00–4.00
Eosinophils ( × 10^9^/L)	0.04	0.00–0.50
Reticulocytes (%)	2.8	0.3–2.5
Reticulocyte count ( × 10^9^/L)	59	20–110
International normalised ratio (ratio)	1.2	0.8–1.2
Activated thromboplastin time (seconds)	27	22–32
Fibrinogen (g/L)	2.8	1.5–4.0
D-dimer (mg/L)	0.12	0.00–0.20
Haptoglobin (g/L)	1.18	0.36–1.95
Lactate dehydrogenase (U/L)	157	100–200
Direct anti-globulin test	Negative	
C-reactive protein (mg/L)	0.6	0.0-5.0
Sodium (mmol/L)	134	135–145
Potassium (mmol/L)	3.8	3.5–4.5
Chloride (mmol/L)	105	101–111
Bicarbonate (mmol/L)	22	22–32
Urea (mmol/L)	17.6	2.5–9.6
Creatinine (μmol/L)	260	55–105
Calcium (mmol/L)	2.15	2.20–2.60
Magnesium (mmol/L)	0.67	0.74–1.03
Phosphate (mmol/L)	1.36	0.80–1.50
Albumin (g/L)	28	35–45
Alkaline phosphatase (U/L)	112	30–120
Alanine transaminase (U/L)	42	7–56
γ-glutamyl transferase (U/L)	203	7–64
Vitamin B12 (pmol/L)	494	140–670
Red cell folate (nmol/L)	3110	>800
Uric acid (mmol/L)	0.66	0.24–0.50

Autoimmune studies were negative including: anti-nuclear, anti-extractable nuclear antigens, anti-neutrophil cytoplasmic, anti-glomerular basement membrane, anti-double stranded DNA antibodies. The serum electrophoresis revealed neither serum paraprotein nor increased free light chains. Serology was negative for hepatitis B, C and HIV. Serum IgA level was elevated at 6.7 g/L (RR, 0.80–4.50 g/L) with normal IgG and IgM levels. Serum complement was low on presentation, with a C_3_ of 0.56 g/L (RR, 0.79–1.52 g/L) and C_4_ of 0.10 (RR, 0.16–0.38 g/L). Blood cultures were negative on two separate occasions.

Urinalysis of a sterile, mid-stream sample confirmed haematuria, with an erythrocyte count >999× 10^6^/L (RR, <13 × 10^6^/L) and leukocyte count of 10 × 10^6^/L (RR, <10 × 10^6^/L). Urine sediment analysis showed dysmorphic erythrocytes and casts, consistent with a glomerular origin. A 24-h urine collection showed a protein excretion of 1.8 g/24 h (protein:creatinine ratio 0.25 g/mmol; RR, 0.00–0.03 g/mmol). Abdominal ultrasound demonstrated mild hepatomegaly with diffuse echogenicity consistent with hepatic steatosis. The kidneys measured 11.1 cm and 11.4 cm, with no hydronephrosis. Urine cytology was negative for malignant cells.

**Fig. 1:**
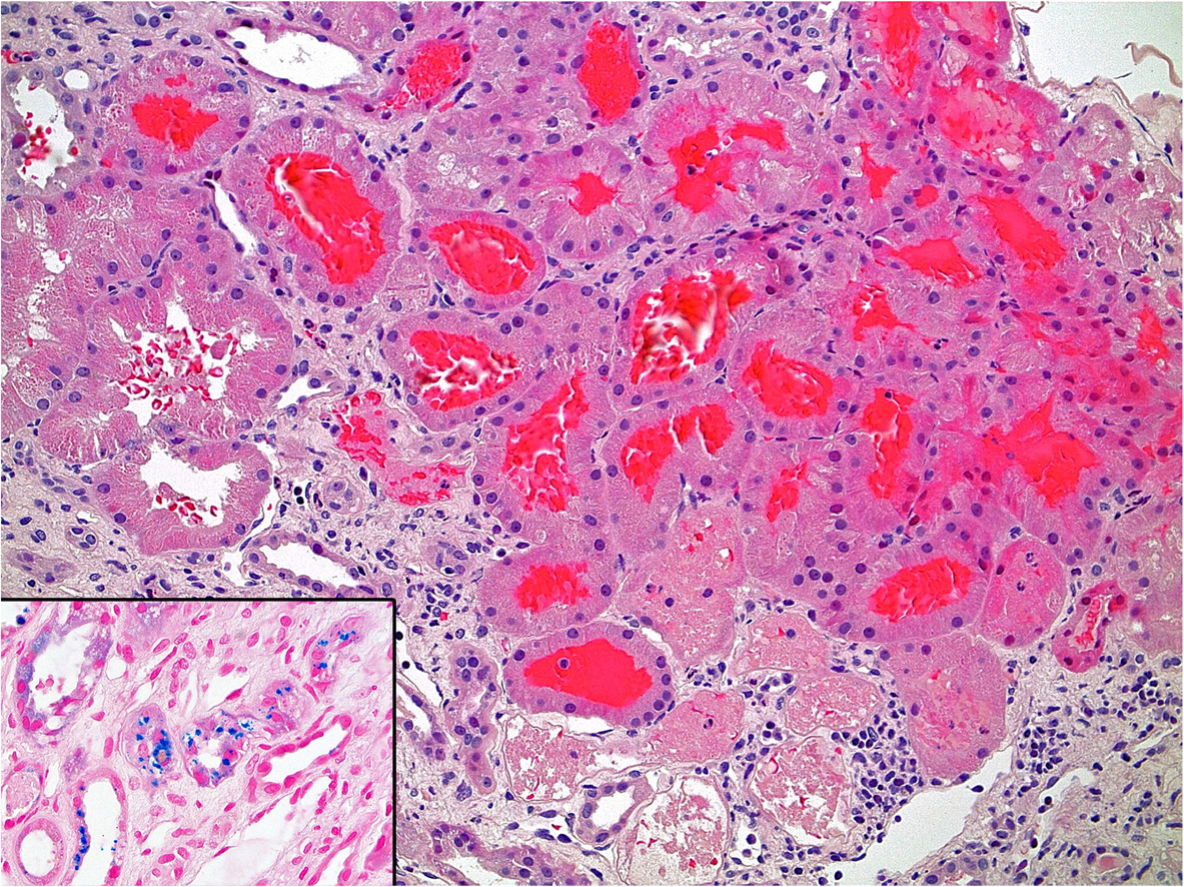
Kidney biopsy showing obstructing tubular red cell casts associated with acute tubular injury (haematoxylin and eosin stain, magnification × 200). Inset: Perl’s stain demonstrating haemosiderin (blue) accumulation within tubular epithelial cells (magnification × 200)

**Fig. 2:**
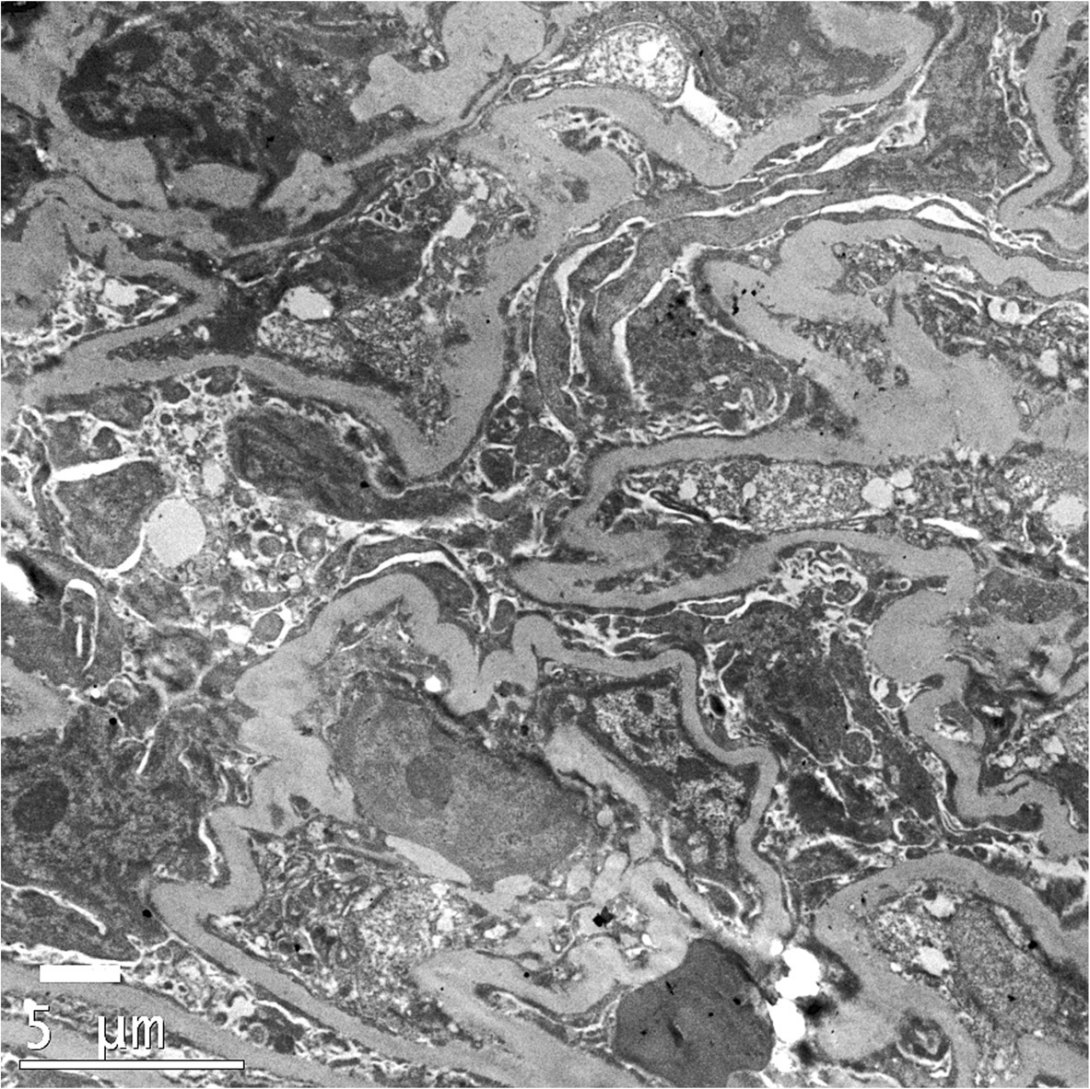
Electron microscopy demonstrating widespread effacement of podocyte foot processes in keeping with a podocytopathy. The glomerular basement shows considerable variation in calibre, with focal areas of attenuation. The mean thickness of the glomerular basement membrane was 251 nm (original magnification × 5000)

Acute glomerulonephritis was suspected and he was treated with intravenous methylprednisolone. An urgent kidney biopsy was performed after platelet transfusion to a count of 102 × 10^9^/L. A bone marrow biopsy was also performed. The kidney specimens demonstrated cortex with 27 glomeruli, of which 6 were globally sclerosed. The principal abnormality was extensive tubular intra-luminal erythrocyte cast formation associated with acute tubular necrosis (Fig. 1). The presence of haemosiderin deposition within tubular cells was suggestive of recurrent glomerular bleeding. There was interstitial fibrosis and tubular atrophy (approximately 30 %), along with a mild inflammatory infiltrate without eosinophils or granulomas. Glomerular capillary loops appeared normal and there were no segmental necrotizing lesions or crescents. Immunoperoxidase stains showed weak, non-specific mesangial staining for IgM (1+) and no significant staining for IgA, IgG, C3, C1q and fibrinogen. Electron microscopy showed thinning of the glomerular basement membrane but no immune complexes. The average glomerular basement membrane thickness was 251 ± 70 nm (range, 125–439 nm), consistent with thin basement membrane disease. This was associated with incomplete pedicel effacement, indicating podocytopathy (Fig. 2). Bone marrow examination showed moderately reduced megakarypoeisis but otherwise normal haemopoeitic function. Cytogenetics and immunophenotyping were unremarkable.

The patient’s macroscopic haematuria ceased after correction of platelet count and intravenous desmopressin given for biopsy. The serum creatinine peaked at 552 μmol/L and subsequently improved. He maintained a good urine output and was discharged for outpatient follow-up. He achieved a best serum creatinine of 250 μmol/L at 6 months, with significant chronic kidney disease (eGFR, 25 ml/min) with mild proteinuria (protein:creatinine ratio, 0.07–0.10 g/mmol). He continued to experience episodes of macroscopic haematuria and AKI although not to the extent experienced on initial admission. His platelet count continued to fluctuate at 26–53 × 10^9^/L. A second bone marrow biopsy 4 months later only showed mildly reduced megakaryopoiesis. A reduced production of thrombopoeitin associated with liver disease and the direct toxic effects of alcohol may be potential factors contributing to the reduced platelet production. Repeat serum complement and autoimmune studies were normal on two separate occasions. After nine months of ongoing alcohol intake, repeat abdominal ultrasound noted hepatic nodularity and small volume ascites. The spleen was borderline enlarged, measuring 14.0 × 7.24 × 6.08 cm, giving a splenic volume of 323 cm^3^. Thus, splenic sequestration-destruction could also be contributing to the thrombocytopaenia. The follow-up ultrasound also noted a reduction in kidney size (right 9.4 cm, left 10.2 cm), associated with an eGFR of 15 ml/min. Due to progression of his chronic kidney disease, he has been worked up for long term haemodialysis with the creation of an arterio-venous fistula.

## Discussion

The differential diagnosis of AKI in the setting of thrombocytopaenia includes infection (e.g. sepsis with disseminated intravascular coagulation, malaria, leptospirosis), malignant hypertension, autoimmune disease (e.g. systemic lupus erythematosus), myeloma, drug side-effect (e.g. ticlodipine) and thrombotic microangiopathy (e.g. haemolytic-uraemic syndrome, thrombotic thrombocytopaenic purpura). In our patient, the clinical, biochemical and serological findings did not support any of these diagnoses. Infection was unlikely given the absence of fever, normal C-reactive protein and sterile blood cultures. There was no laboratory evidence of haemolysis or neurological abnormality to suggest thrombotic microangiopathy. Rarely, thrombocytopaenia and glomerular disease can result from mutations in the myosin heavy chain 9 (*MYH9*) gene. The *MYH9*-related disorders are also associated with large platelets (macrothrombocytopaenia), leukocyte inclusion bodies and sensori-neural hearing loss. Our patient had no family history of renal disease or thrombocytopaenia and was not hearing impaired. He had morphologically normal leukocytes and platelets on a standard blood film. The mean platelet volume was 8.6 fL (RR, 6.5–12.0 fL), which argues against macrothrombocytopaenia. His thrombocytopaenia was also a recent development, having had a platelet count of 230 × 10^9^/L four years previously, suggesting an acquired rather than inherited abnormality.

The association between anticoagulation and haematuria is well recognized and may result in AKI through different mechanisms [1]. A number of case reports have suggested that an underlying glomerular abnormality may predispose to warfarin-related nephropathy, including lupus nephritis [3], focal segmental glomerulosclerosis [4], IgA nephropathy [5] and thin basement membrane disease [6]. In the original case series of nine patients reported by Brodsky et al. on warfarin-related nephropathy, five patients had mild glomerular immune deposition (of which three were IgA on immunofluorescence) and one had focal segmental glomerulosclerosis. However, the status of the glomerular basement membrane was not mentioned in that paper [2]. In the absence of coagulopathy, macroscopic haematuria with AKI is mostly reported with IgA nephropathy. Other causes of glomerular haematuria such as Alport’s syndrome and proliferative glomerulonephritides (e.g. lupus, vasculitis) have not been clearly associated with macroscopic haematuria-associated AKI [1].

We report a patient with an acquired thrombocytopaenia precipitating glomerular haematuria and tubular injury, who was subsequently discovered to have thin basement membrane disease on renal biopsy. The mechanism of AKI is similar to that described for warfarin-related nephropathy and IgA nephropathy. Typically, there is acute tubular injury with prominent obstructive erythrocyte casts [1, 2]. The incidence of macroscopic haematuria due to severe thrombocytopaenia in adults is unclear. In a paediatric retrospective series of 332 children with immune thrombocytopaenia over a 10-year period, 17 % experienced at least one episode of major haemorrhage, which included a subgroup with macroscopic haematuria (6 episodes of macroscopic haematuria, 17 episodes of bleeding from multiple sites which may include haematuria). Major haemorrhage mostly occurred in patients with platelet counts <20 × 10^9^/L. Only 13 % of events occurred with platelet counts of 20–75 × 10^9^/L. The nature of the bleeding or presence of pre-existing kidney disease was not specified [7]. In a prospective observational study of 169 neonates with platelet counts <60 × 10^9^/L, haematuria was detected in 40 % by dipstick or microscopy [8]. These paediatric studies suggest that thrombocytopaenia-associated macroscopic haematuria is rare compared to microscopic haematuria. However, the source of the haematuria has not been clearly ascertained in these studies (glomerular or urological).

In warfarin-treated patients, haematuria may be non-glomerular (urological) rather than glomerular (warfarin-related nephropathy), with the risk of haematuria much higher in over-anticoagulated patients and may be transient [9]. In thrombocytopaenic patients, there are certainly case reports demonstrating ureteric bleeding, with or without associated stone disease [10, 11]. Histologically demonstrable extravasation of erythrocytes from the bladder capillaries, manifesting as haematuria with diffuse submucosal petechiae on cystoscopy has been described in a patient with immune-mediated thrombocytopaenia (platelet count 33 × 10^9^/L) [12]. The evidence for glomerular haematuria in thrombocytopaenic patients, however, is lacking. Thus, this is a unique case report which histologically demonstrates glomerular haematuria-associated AKI in the setting of severe thrombocytopaenia.

There is also strong epidemiological evidence that AKI is associated with the development and progression of chronic kidney disease. This has also been suggested by studies of warfarin-related nephropathy [13]. The concern of recurrent AKI begs the question of how aggressive clinicians should treat severe thrombocytopaenia in patients with an underlying risk factor for glomerular haematuria. Thin basement membrane disease has traditionally been considered a relatively benign condition but recurrent episodes of AKI related to macroscopic haematuria may be considered a risk factor for chronic kidney disease progression and end-stage renal failure.

## Conclusions

Haematuria in the setting of severe thrombocytopaenia may be glomerular or non-glomerular in origin. Given the rarity of AKI from glomerular haematuria with platelet counts >20 × 10^9^/L, an underlying glomerular abnormality such as thin basement membrane disease should be considered. Heavy glomerular bleeding may result in recurrent AKI and progression of chronic kidney disease in patients with thin basement membrane disease.

## Consent

Written informed consent was obtained from the patient for publication of this case report and any accompanying images. A copy of the written consent is available for review by the Editor of this journal.

## Abbreviations

AKI: acute kidney injury
eGFR: estimated glomerular filtration rate
RR: reference range
